# Associations between fitness levels and self-perceived health-related quality of life in community - dwelling for a group of older females

**DOI:** 10.1186/s12889-020-08473-3

**Published:** 2020-08-17

**Authors:** Ferenc Ihász, Nikolett Schulteisz, Kevin J. Finn, Krisztina Szabó, Judit Gangl, Dóra Nagy, Pongrác Ács, András Oláh

**Affiliations:** 1 Department of Physical Education and Sport Sciences, Faculty of Pedagogy and Psychology, University of Eötvös Lóránd, Szombathely, Hungary; 2 Faculty of Health Sciences, University of Pécs, Pécs, Hungary; 3 Faculty of Health Sciences, Doctoral School of Health Sciences, University of Pécs, Vörösmarty u. 4, Pécs, H-7621 Hungary; 4 School of Nutrition, Kinesiology, & Psychological Sciences, College of Health, Science and Technology, University of Central Missouri, Warrensburg, MO USA

**Keywords:** Fullerton fitness test, Self-motivation, Motor skills, Physical fitness

## Abstract

**Background:**

For older adults perceived quality of life has been linked to the ability to accomplish everyday tasks, a functional capacity which is thought to be based upon physical fitness. Although there is a relationship between physical activity and quality of life in older adults, the fitness of older adults and its relationship to quality of life needs more investigation. Therefore, the purpose of this study was to examine the associations between self-reported health-related quality of life and physical fitness in community-dwelling older females.

**Methods:**

A cross-sectional study between four different age groups in retirement villages from two different places of the southern and western region of Hungary, among 173 women between the ages of 58 and 94 years old. We measured physical fitness using the Fullerton Test protocol and self-perceived health quality of life using the Short-Form Health Survey.

**Results:**

Group means were different in six-minute walk distance, handgrip strength, and arm curls. The youngest group of females had higher scores of fitness in these categories as compared to the oldest grouping of women. Quality of Life were also difference across age groupings although not linear across the four age categories. Moderate level positive relationship was evident between perceived physical function and certain categories of physical fitness.

**Conclusions:**

Sociability and self-motivation has a leading role in quality of life in elder population. It is worth putting a lot more emphasis into continuous cultural, social and most importantly into physical activity programs for elderly.

## Background

The demographic landscape of the world is rapidly changing, with older adults representing the fastest growing segment of the populations in many areas in the world [1]. It is well-known that advancing age is associated with predictable sensory, motor and cognitive changes, many of which potentially impact an older person’s ability to function effectively in society [2–4].

At an advanced age, structural and functional deterioration occurs in most physiological systems, even in the absence of discernible disease. These age-related physiological changes affect a broad range of tissues, organ systems and functions, and can cumulatively impact successes and activities of daily living (ADL) [5]. The combined effect of mobility and cognitive capacity as a risk factor of institutionalization among initially community-dwelling people aged 75–80 years provides evidence that the risk for dependency is 4.9 times greater for those who had some mobility limitation or cognitive deficit than for those with no limitation [6].

Kopkáné Plachy J et al. investigated 45 elderly women divided into three groups: one of them did a half year of physical activity sessions three times per week (Training group), the other had two physical exercise sessions and one group discussion about healthy lifestyle per week (Mental group) and a Control group which wasn’t involved in the activities. They aimed to assess whether the two different activity programmes had positive effects on health dimensions by analysing mental health status (SF-36) and fitness status (FFFT) results and measuring bone density. The results showed significant differences between the Control and both the Training and Mental groups which contributes to better health status of the participants [7].

In one study, body composition of people aged 65–99 years with functional and cognitive impairment was examined in residential care facilities and showed that sarcopenia may lead to significant problems in daily life [8]. Women have lower fat-free mass (FFM) and higher fat mass (PBF%), inversely related to age, than men. Physical activity has been consistently associated with quality of life in older adults [9]. Increased physical activity (PA) protects against functional decline, health disease, diabetes, bone fracture and falling, and also improves sleep and quality of life for older adults [10–12]. In addition, higher fitness levels have been shown to show lower prevalence on mortality associated with cardiovascular disease [13, 14]. Fitness is very important for those in their senior years. Older adults need to have adequate strength, flexibility, and endurance to accomplish everyday tasks [15]. Assessing these components of fitness can detect weaknesses which can be treated before causing serious functional limitations.

The purpose of this study was to examine the associations between self-reported health-related quality of life (HRQoL) and physical fitness in different age groups in community-dwelling older females. It is hypothesized that as the older study participants will score lower on physical fitness tests. Also it is hypothesized that those participants with higher physical fitness scores will report higher scores in the HRQoL.

## Methods

A cross-sectional study was conducted from the long-term residential institution in two different facilities of the Hungarian regions (Southern Hungary, Pécs, Western Hungary, Győr). In both locations, these institutions operate in a similar manner in the research methodology. In a preliminary review, the average age, motor performance, body composition and quality of life of the residents of the two retirement homes are not statistically different. Thus, it was decided to combine the two samples. No significant difference was found in the quality of life of the people living in the two cities.

All residents were invited to a preliminary meeting in which they were informed about the nature, benefits, and risks of the study. Those who agreed to participate in this study provided written informed consent, consistent with the Helsinki Declaration and completed a health history questionnaire. The exclusion criteria included all physical or psychological conditions that could interfere in the capacity to undertake the tests requested and the use of medication that might influence functional performance or the interpretation of the results. Medication consumption was assessed by consulting the computerized records of each of the participant’s family physicians. It was obligatory for the individual records of each patient to state all of the medication prescribed by the family physician. Only medication consumed regularly was considered, such as that for chronic diseases. Self-medication was not considered in this evaluation. From this recruitment process, 173 women aged between 58 and 94 years (79.6 ± 8.7 years) were included in the study. These participants were kept on similar diets in terms of caloric and nutritional intake, controlled by a nutritionist, and any medication dosages, including salicylate and statins, remained unchanged during the study.

Body height (BH) was measured to the nearest 0.1 cm with a stadiometer (Seca 208 Bodymeter). Upper arm, calf, and thigh circumferences were measured with anthropometric tape recorded to the nearest 0.1 cm. In addition, body mass (BM), fat mass (FM), were determined using the InBody 720, an octopolar bioimpedance analyser (Biospace, Seoul, Korea). The validity of this bioimpedance for body composition has been previously documented [16].

Physical fitness was evaluated using the Fullerton Fitness Test [17, 18]. This senior fitness test was developed as part of the LifeSpan Wellness Program at Fullerton University. It is a simple, easy-to-use battery of test items that assess the functional fitness of older adults. The test manual describes easy to understand instructions to measure aerobic fitness, strength and flexibility using minimal and inexpensive equipment [19]. The test items included hand-grip strength, arm curls, 30 s chair stand, upper and lower body flexibility, and the six-minute walk.

The hand-grip strength was isometrically measured using an electronic hand-grip dynamometer (Takei, TKK 5101 Grip-D) as suggested elsewhere [20]. The subject held the dynamometer in the dominant hand hanging down by his or her side and was asked to squeeze using maximum force. The best score obtained in three trials, with approximately a 2-min rest between trials, was recorded for each subject. For this study, the best score for each hand was used in the analyses.

The arm curl test was used to measure upper body endurance [21]. Subjects performed seated a biceps curls without bending the trunk forward for 30 s with 2.3-kg dumbbells. The total number of arm curl repetitions was the score used for the analyses.

The 30-s Chair Stand was used to measure lower limb muscle strength and mobility. The score equals the number of rises from a chair in 30 s with arms folded across the chest [22].

Upper body flexibility was assessed using the “back scratch” task performed in the standing position. The dominant hand was placed over the ipsilateral shoulder with the fingers outstretched downwards as far as possible. The other hand was placed behind the back with the palm directed to the outside and the fingers outstretched upwards to try to hold on to the fingers of the other hand. The testing technician instructs the participant, how to position the hands so that the middle fingers are possibly close. Catching or pulling participant’s fingers was not allowed. A 30-cm ruler was used determine the distance measured between the middle fingers of the hands. If the fingers overlap, the value is positive “+”, if otherwise, it is negative “-”. The measure was reported within a precision of 0.5 cm.

Lower body flexibility was assessed using the “chair sit”, determining primarily the elasticity of popliteal tendons. The participant sat on the edge of the chair. One leg was resting with the whole foot on the ground. The second, dominant, was outstretched, resting with the heel on the ground, the foot flexed at a right angle. The trial involves flexion forward maintaining the vertebral spine as straight as possible with the head positioned along the vertebral axis. The arms are outstretched forward and the hands placed on each other (the middle fingers at the same height). The participant tries to touch the toes with the fingers. The reach of the flexion should be maintained for 2 s. During the test, instructions were provided indicating that rapid, powerful movements should be avoided and the pain threshold should not be exceeded. The distance measured between the middle finger and the first toe is the value obtained from the trial. A positive value “+” indicates that the fingers crossed the toe line, a negative value “-” indicates that the fingers did not cross the toe line. The value was measured at a precision of 0.5 cm.

Aerobic fitness was evaluated using the six-minute walk test (6MWT), which is a physical performance test widely used in research [23, 24], especially to obtain valid measures of submaximal aerobic endurance in older adults [25]. This test measures the distance covered when subjects are instructed to walk as quickly as they can for 6 min. Walks were conducted on a flat 50-m rectangular course, marked off in five-meter segments. If necessary, subjects were allowed to stop and rest [23–27].

Subjective health-related quality of life was assessed using the perception of health on the HRQoL SF-36 [28]. This instrument is a relatively simple and brief questionnaire developed to measure generic health status and quality of life. It was demonstrated that this questionnaire is suitable for community-dwelling older adults when administered by personal interview [29]. The SF-36 comprises eight health scales: physical functioning (PF; ten items), role limitations due to physical problems (RP; four items), bodily pain (BP; two items), general health (GH; five items), vitality (VT; four items), social functioning (SF; two items), role limitations due to emotional problems (RE; three items), and mental health (MH; five items). There is also a single separate item that is used to assess any change in health from the previous year. The SF-36 was administered by interview, and scores were calculated using the methods set out by Ware et al. [30]. The scores range from 0 to 100, with higher scores indicating better functional health and well-being.

The study participants were grouped based upon age nearest each decade of 60, 70, 80, and 90 years. Descriptive analyses were conducted to determine group means and standard deviations while an Analysis of Variance (ANOVA) was used to test for group differences. The alpha was set at the 95th level of confidence (*P* < 0.05) for significance. Significant group differences were tested using post-hoc comparisons. Finally, ranked order correlations were conducted between scores on the quality of life health scales and the measures of physical fitness.

## Results

Table 1 listed the means (and standard deviations) for body size, girth sizes, and body composition measure for each group. The ANOVA indicated significant differences in body mass between groups.

**Table 1:** Comparison of anthropometric, body composition characteristics, based on age group (1–4 groups), in (60–90) years old women

	*n* = 35 (1 group)	*n* = 30 (2 group)	*n* = 64 (3 group)	*n* = 36 (4 group)	
Variable	Mean ± SD	Mean ± SD	Mean ± SD	Mean ± SD	P
DA (years)	60.00 ± 5.05*	71.40 ± 2.43*	80.97 ± 2.73*	89.34 ± 2.41*	(1–2); (1–3); (1–4); (2–3); (2–4); (3–4)
BW (kg)	62.16 ± 12.79**	78.44 ± 21.51**	67.57 ± 14.15*	59.99 ± 10.29**	(1–2); (1–3); (1–4); (2–3); (2–4); (3–4)
BH (cm)	161.33 ± 12.21	160.76 ± 7.23	159.17 ± 7.32	157.22 ± 8.47	NS
TC (cm)	45.25 ± 6.99**	47.65 ± 7.42	46.05 ± 6.83	41.79 ± 6.23**	(1–4); (2–4); (3–4)
CC (cm)	34.75 ± 3.51	35.96 ± 4.85	35.12 ± 4.24	33.31 ± 3.99	NS
UaC (cm)	29.71 ± 5.53**	28.15 ± .,96	27.72 ± 3.56	26.30 ± 4.18**	(1–4); (2–4)
PBF%	26.53 ± 3.27**	36.85 ± 9.40**	36.52 ± 8.86**	31.61 ± 10.04*	(1–2); (1–3); (1–4); (2–4)

*Abbreviations*: *DA* decimal age (year), *BW* body weight (kg), *BH* body height (cm), *TC* thigh circumference, *CC* calf circumference, *UaC* upper arm circumference, *PBF%* relative body fat

*P* < 0.05* *P* < 0.01**

As regards for the average age of the body weight groups, the highest values were found among 70-year-olds and the lowest for 90-year olds. There was a significant difference between body mass in group 1 (youngest) and group 2 as well as groups 3 (older) and group 4 (oldest). A significant increase in body mass was found between the youngest and next decade age grouping of females. Also, there was a significant decline in body mass between group 2, group 3 and group 4. The smallest body height (BH) averages were in the 90-year-olds, close to ~ 4 cm in comparison on average to the 60-year-olds however these means were not significant.

For the limb circumferences, the thigh and upper arm measures were significantly different between groups. The difference between the group averages of the thigh circumference is evident between the 60 and 90 (1 and 4) and 70 and 90 (2 and 4) age range showing a decline in the group means. For the upper arm circumference, a similar decline in group means were evident between the younger groups (1 and 2) in comparison to the oldest group (group 4). The relative body fat (PBF%) of 60-year-old at 26.53 ± 3.27%, was significantly lower than the other age groups.

Table 2 lists the group means, standard deviations, and ANOVA results for fitness tests separately. The handgrip averages are significantly different for each group in both right and left hands. As the age of the group increased, the handgrip strength declined across groups. The arm curl test showed significant differences between the youngest and oldest groupings with less repetitions in the oldest group. No differences were evident between arm curl in the first three groups of participants. The measures of upper body (back stretch) and lower body (chair sit and reach) flexibility were not different between groups. Results from the walking test (6MWT) showed significant difference in the distance (m) travelled but only between the youngest and oldest groups (315.42 ± 194.35vs.188.58 ± 104.93); *p* < 0.01.

**Table 2:** Comparison of fitness tests between groups

	*n* = 35 (1 group)	*n* = 30 (2 group)	*n* = 64 (3 group)	*n* = 36 (4 group)	
Variable	Mean ± SD	Mean ± SD	Mean ± SD	Mean ± SD	P
Handgrip r. s.) (kg)	24.51 ± 10.18*	22.27 ± 8.41*	18.12 ± 5.62*	16.62 ± 6.88*	(1–2); (1–3); (1–4); (2–3); (2–4)
Handgrip l. s. (kg)	22.59 ± 7.75**	20.31 ± 7.24**	16.93 ± 5.72*	15.11 ± 6.71**	(1–2); (1–3); (1–4); (2–3); (2–4)
Back Scratch (BS), (cm)	−12.75 ± 16.13	− 16.48 ± 1.,65	− 20.36 ± 16.34	−16.25 ± 13.72	NS
Arm Curl, (AC) (reps)	20.56 ± 3.93**	19.31 ± 6.16	18.04 ± 5.64	17.13 ± 6.56**	(1–4)
Chair sit & r., (Csr) (cm)	1.93 ± 3.10	−1.54 ± 10.18	−2.66 ± 10.54	−1.05 ± 8.98	NS
30-s Chair stand (30-s Cs) (amount)	11.21 ± 6.91	12.25 ± 4.44	10.65 ± 4.85	10.28 ± 4.14	NS
6MWT (m)	315.42 ± 194.35**	243.21 ± 113.55	228.90 ± 132.52	188.58 ± 104.93**	(1–4)

*Abbreviations***:** *Hg rs.* Handgrip right side (kg), *Hg ls.* Handgrip left side (kg), *BS* Back Scratch (cm), *AC* Arm Curl (cm), *Csr* Chair sit & r. (amount), *30-s Cs* 30-s Chair stand (amount), *6MWT* 6 min walking test (m)

*P* < 0.05* *P* < 0.01**

Table 3 lists the group means and standard deviations between groups in responses to the Quality of Life for the eight scales of this 36-item questionnaire. With the exception of bodily pain and social functioning, all the other life quality characteristics measured show significant differences by age groups. In the 70 (group 2) and 80 (group 3) age groups, general health (GH) and vitality (VT) was significantly lower than the 60 (group 1) and the 90 year- old grouping. Perception of physical functioning varied between age groups. The 70-age group has lower perception of physical functioning than the other three groups whereas the oldest group reported the highest score in this category. The perceived role limitations due to physical limitations were the highest in the oldest group followed second in rating but the 70-year-old grouping.

**Table 3:** Comparison of the quality of life between groups

	*n* = 35 (1 group)	*n* = 30 (2 group)	*n* = 64 (3 group)	*n* = 36 (4 group)	
Variable	Mean ± SD	Mean ± SD	Mean ± SD	Mean ± SD	P
Phys. func. (PF)	60.62 ± 37.73*	49.35 ± 34.46*	61.17 ± 25.15*	64.72 ± 26.77*	(1–2); (1–3); (1–4); (2–3); (2–4); (3–4)
Role of Phys. probl. (RP)	81.25 ± 29.58**	87.09 ± 30.18**	78.30 ± 34.79*	88.88 ± 28.31**	(1–2); (1–3); (1–4); (2–3); (2–4); (3–4)
Bodily pain (BP)	66.87 ± 20.43	63.45 ± 22.99	58.91 ± 24.50	61.83 ± 19.91	NS
Gen. health (GH)	62.25 ± 1.,45**	52.38 ± 25.70**	58.47 ± 19.31**	65.91 ± 18.96**	(1–2); (1–3); (1–4); (2–3); (2–4); (3–4)
Soc. func. (SF)	85.15 ± 21.99	87.50 ± 23.27	84.19 ± 19.27	88.54 ± 17.51	NS
Vitality. (VT)	74.37 ± 18.84**	56.12 ± 24.82**	58.97 ± 23.60**	69.44 ± 2.16**	(1–2); (1–3); (1–4); (2–3); (2–4); (3–4)
Role of Emos. prob. (RE)	89.58 ± 15.95**	88.17 ± 26.59**	73.52 ± 36.67**	86.11 ± 31.24*	(1–2); (1–3); (1–4); (2–3); (2–4); (3–4)
Ment. health. (MH)	55.50 ± 18.23**	62.58 ± 23.87**	61.35 ± 18.84**	63.77 ± 14.90**	(1–2); (1–3); (1–4); (2–3); (2–4); (3–4)

*Abbreviations*: *PF* physical functioning, *RP* role limitations due to physical problems, *BP* bodily pain, *GH* general health, *VT* vitality, *SF* social functioning, *RE* role limitations due to emotional problems, *MH* and mental health

*P* < 0.05* *P* < 0.01**

Mental health and role limitations due to emotional problems showed significant differences between groups. Lower scores are reported in the youngest group (60-year olds) for mental health but highest in the scale for the role limitations due to emotional problems. The highest score for mental health were in the oldest age group (90-year olds).

Table 4 listed the correlation coefficients from each of the self-reported HRQoL scales and the scores from the six-minute walk distance, handgrip strength, and 30s chair stand. The findings indicated that positive moderate relationships existed between the participants’ perception of physical functioning and the six-minute walk (rho = 0.368) and the 30s chair stand (rho = 0.370). A weaker but significant relationship was evident between the perceived role limitations due to physical problems and grip strength (rho = 0.269 for right hand, rho = 0.265 for left hand). Other notable significant relationships existed between vitality and the handgrip strength on the right hand (rho = 0.369). Bodily pain and the six-minute walk distance were related (rho = 0.277) also.

**Table 4:** Spearman’s rho correlation coefficients between each self-reported HRQoL scales and each physical fitness variable

	6MWT	Handgrip right side	Handgrip left side	30-s Chair Stand
PF	0.368*	0.150	0.269*	0.370*
RP	−0.032	0.269*	0.265*	0.110
BP	0.277*	0.138	0.031	−0.029
GH	0.122	0.051	0.041	0.023
VT	0.089	0.369*	0.146	−0.001
SF	− 0.099	−0.072	− 0.098	−0.142
RE	0.009	0.147	0.152	0.064
MH	0.040	−0.123	−0.112	− 0.028

*Abbreviations*: *PF* physical functioning, *RP* role limitations due to physical problems, *BP* bodily pain, *GH* general health, *VT* vitality, *SF* social functioning, *RE* role limitations due to emotional problems, *MH* mental health

*P* < 0.05*

**Fig. 1:**
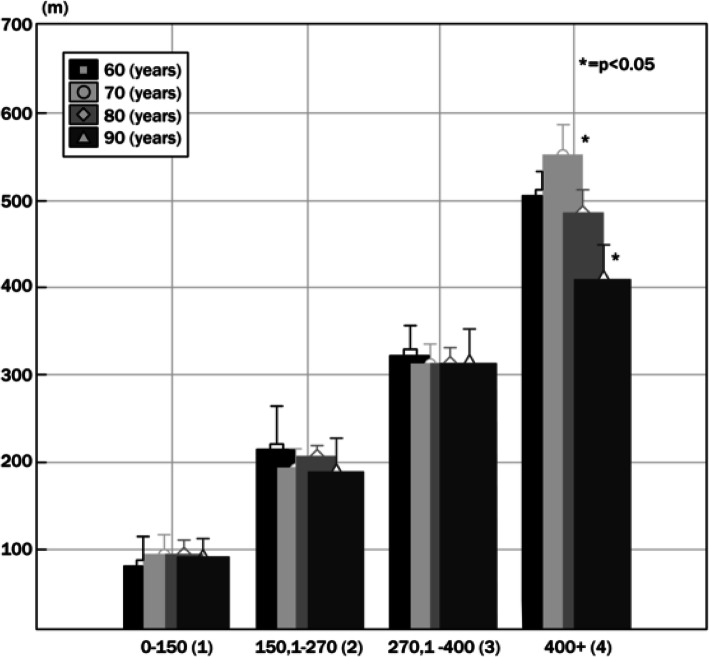
Results of the accomplished distance (meter) and age (60–90) year based on performance

**Fig. 2:**
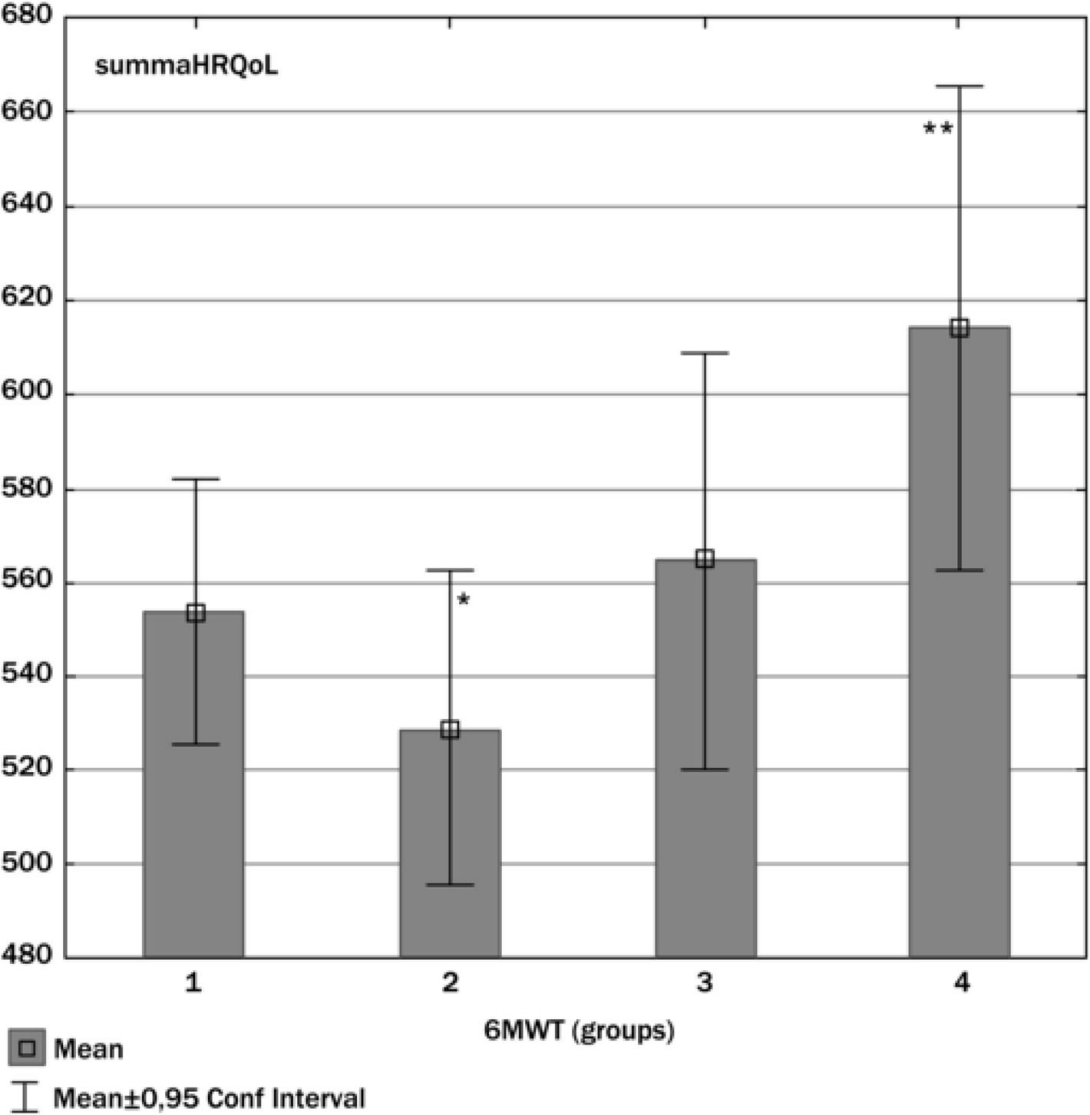
Summarized self-reported HRQoL domains (480–680) in relation to distance travelled in the 6-min walking test (6MWT)

Figure 1 examines the walking distance of each age group based on the performance groups. Only in the fourth (4) group was there a significant difference in distance (meters) between the 70-year-old (410 ± 78) and the nighty-year-old (553 ± 105) females (*p* < 0.05). Figure 2 illustrates the summarized self-reported HRQoL domains (480–680) in each of the groups of the distance travelled in the 6-min walk test. The participants who only walked in group 1 (90.3 ± 30.03 m) and group 2 (198 ± 32.8 m) presented lower summarized self-reported HRQoL domains. The distance travelled increased, in group 3 (314.3 ± 26.8), group 4 (493.7 ± 65.1) had highest summarized self-reported HRQoL domains. In terms of self-reported HRQoL domains, differences were significant (*p* < 0.01) between group 2 and group 3, between group 2, group 4 and group 3 and group 4.

## Discussion

The main findings of this study show that the way an older physically independent individual reports some aspects of his HRQoL is related to physical fitness. The relationship between physical fitness and health is complex, and there is no single study or instrument able to simultaneously elucidate the mechanisms for all the core dimensions that have been identified as being associated with health and functioning (e.g., living arrangements, life styles) [31]. Thus, it is possible that other unmeasured variables can explain the association between physical fitness and HRQoL.

In one study researchers found, that the quality of life of the elderly is influenced by physical and functional aspects, as well as sociological and psychological aspects, with mood interpreted as the set of emotional states experienced by each person in their daily lives. The emotional dimension is connected to various feelings, whether negative (tension, anger, fatigue, and depression) or positive (self-esteem, vigor and wellbeing) and is therefore associated with the individual’s health and quality of life [32]. Barthalos and Bognar et al. examined differences in Quality of Life, fitness and body composition between elderly, chosen from twilight homes and clubs for retired people. Examining QOL’s main items, they found self-efficiency and physical activity depended on health and social status. Self-perception and attitudes toward death, autonomy, and sociability were different in the two groups and played an important role as a QOL indicator and an outcome of physical activity [33]. According to another study from these researchers, personal relationship, physical environment, meaning in life, and health satisfaction is considered to be the most important components of quality of life by the participants [34].

Considering the possible limitations and the strengths mentioned above, the findings of this research demonstrated through objective measurements of physical fitness that among relatively healthy community-dwelling older individuals, lower levels of physical fitness were associated with lower self-reports of several domains of HRQoL as measured by the SF-36. In this study, the age range of the tested sample varies between 58 and 92 years, and therefore based on age, four groups were created and used to compare their results. The difference in the relative fat mass of the PBF averages were constantly increasing. With the exception of the 60-year-olds, all the examined groups averages of PBF report on obese samples. This finding is predictable in this age range, given that over the years there is a decrease in lean mass and an increase in fat mass but is not acceptable, given that this is associated with an increase in the number of comorbidities. One of the reliable predictors of general muscle power is the analysis of symmetrical handgrip. In this particular sample, this performance decreases with age. The difference between the averages in groups 1 to 4 is ~ 8 kg. To our surprise, a real difference in the average of 30-s chair stands between age groups were not found.

Corroborating with our results, Park, S.W. et al. investigated long-term decreases in lean body mass and strength in older adults with and without T2D and the results showed significant associations between aging and muscle loss and muscle quality [35]. Nevertheless, the decreases in lean body mass and quality in both limbs (upper and lower limb) of T2D showed significant reductions in lean body mass of the lower limbs. The possible explanation for the declines in lean body mass and strength might be peripheral artery disease that is a common comorbidity of T2D and could be associated with muscle weakness and atrophy that affects upper and lower limbs.

Significant difference in the travelled distance during the walking test (6MWD) was only found between the 60 and 90-year-olds. Compared to the international average, the travelled distance we have measured is nearly 50% less in every age groups. The 6 min distance is a more objective test of a patient’s functional capacity, requiring the patient to walk in a reproducible environment. The 6MWD has been shown in studies to have good prognostic value in the different subsets of heart failure (HF) patients. In a study of about 200 patients with mild to moderate HF, 6MWD was a strong predictor of mortality. Castel et al. showed that in patients with moderate to severe HF receiving cardiac resynchronization therapy, 6MWD was found to be an independent predictor of mortality [36]. Six-minute walk distance also strongly predicted mortality and HF rehospitalisations in patients hospitalized for acute HF.

Favourable body composition and maintaining motor skills (general muscle strength, dynamic balance, joint flexibility, and aerobic capacity) could make a significant contribution to maintain an independent, good life quality (or well-being). The findings of this study support the role of self-efficacy in the relationship between physical fitness and HRQoL as well as an expanded HRQoL model including both health status indicators and global HRQoL. These findings further suggest future physical activity promotion programs should include strategies to enhance self-efficacy, a modifiable factor for improving HRQoL in this population.

## Conclusion

The findings from this cross-sectional study of physical fitness and self-reported perception of quality of life in a group of older women in community living dwellings revealed that, the levels of physical fitness reflective of aerobic capacity, and muscular strength were significantly lower in the oldest group as compared to the younger groups. No differences were evident in upper and lower body flexibility or the ability to perform repetitive chair stands. The perception of quality of life differed between age groups but not in a linear fashion. The relationship between physical fitness and quality of life was moderately and positively related in areas of perceived physical functioning, role limitations due to physical problems, and vitality.

One important piece of information in the study is that the 6 -min walking test performance is in many cases independent from age and that the oldest group performed well, even the youngest (Fig. 1). Significant difference in the traveled distance during the walking test (6MWD) was only found between the 70 and 90-year-olds. Compared to the international average, the traveled distance we have measured is nearly 50% less in every age group.

Further studies in this area are needed to determine the impact of an active lifestyle on functional fitness (balance training, resistance training). Since the relationship between physical fitness and quality of life is moderately related, changes in measures and training aims would allow to test for a cause-and-effect response. Future studies are planned to increase the number of elements and to conduct a longitudinal study combined with intervention.

## Data Availability

The datasets used and/or analysed during the current study are available from the corresponding author on reasonable request.

## Abbreviations

DA: Decimal age (year)
BW: Body weight (kg)
BH: Body height (cm)
TC: Thigh circumference
CC: Calf circumference
UaC: Upper arm circumference
PBF%: Relative body fat
Hg rs: Handgrip right side (kg)
Hg ls: Handgrip left side (kg)
BS: Back Scratch (cm)
AC: Arm Curl (cm)
Csr: Chair sit & r. (amount)
30-s Cs: 30-s Chair stand (amount)
6MWT: 6 min walking test (m)
PF: Physical functioning
RP: Role limitations due to physical problems
BP: Bodily pain
GH: General health
VT: Vitality
SF: Social functioning
RE: Role limitations due to emotional problems
MH: And mental health
